# Effectiveness of Magnetic Stimulation in the Treatment of Urinary Incontinence: A Systematic Review and Results of Our Study

**DOI:** 10.3390/jcm10215210

**Published:** 2021-11-08

**Authors:** David Lukanović, Tina Kunič, Marija Batkoska, Miha Matjašič, Matija Barbič

**Affiliations:** 1 Department of Gynecology, Division of Gynecology and Obstetrics, Ljubljana University Medical Center, 1000 Ljubljana, Slovenia; tina.kunic@kclj.si (T.K.); marijabatkoska@yahoo.com (M.B.); matija.barbic@guest.arnes.si (M.B.); 2 Center for Social Informatics, Faculty of Social Sciences, University of Ljubljana, 1000 Ljubljana, Slovenia; miha.matjasic@fdv.uni-lj.si; 3 Department of Gynecology and Obstetrics, Faculty of Medicine, University of Ljubljana, 1000 Ljubljana, Slovenia

**Keywords:** urinary incontinence, treatment, magnetic stimulation

## Abstract

Urinary incontinence (UI) is becoming an increasingly common health problem. UI treatment can be conservative or surgical. This paper focuses on the effectiveness of magnetic stimulation (MS) in the treatment of UI. We performed a systematic review in order to combine and compare results with results from our clinical study. A clinical prospective non-randomized study was carried out at the Ljubljana University Medical Center’s Gynecology Division. It included 82 randomly selected female patients, irrespective of their UI type. The success rate of using MS in treating UI was based on standardized ICIQ-UI SF questionnaires. Patients completed 10 therapy sessions on MS, and follow-up was performed 3 months after the last therapy session. UI improved after treatment with MS. The ICIQ-UI SF score improved in patients regardless of the type of UI. However, the greatest decrease in post-treatment assessment ICIQ-UI SF scores was seen in patients with stress urinary incontinence (SUI). Based on the findings described above, it can be concluded that MS is a successful non-invasive conservative method for treating UI. Future studies are necessary, all of which should include a large sample size, a control group, an optimal research protocol, pre-treatment analyses, standardization, and longer follow-ups.

## 1. Introduction

Uncontrolled leakage of urine, or urinary incontinence (UI), is a pelvic floor dysfunction found in all age groups [1]. UI has been used as a term since 2010 for any complaint of involuntary loss of urine, as per the definition by the International Urogynecological Association (IUGA) and the International Continence Society (ICS) joint report on the terminology for female pelvic floor dysfunction [2]. Patients have varied symptoms and signs, and they cite a wide range of problems, from mild to disabling [2,3,4]. The etiology of UI is multifactorial because risk factors include age, pregnancy, and childbirth (multiparous women), pelvic floor injury during vaginal delivery, pelvic surgery, menopause (due to decreased estrogen secretion), hysterectomy, increased body weight, lack of physical activity, urinary tract infections, chronic cough, prolonged heavy lifting, congenital weakness of connective tissue, and chronic constipation [2,4,5].

According to anatomical criteria, UI is divided into urethral and extra-urethral. Clinically, it is divided into absolute and relative UI [6]. Several types of relative UI are known, and they are divided by the basic pathophysiological mechanisms that cause their onset. They are roughly divided into stress UI (urinary incontinence due to pressure or upon exertion, SUI), urgency UI (urgency urinary incontinence, UUI), mixed UI (with characteristics of stress and urgency UI, MUI), and overflow UI (involuntary release of urine due to an overfull bladder). In practice, however, the borders between different UI types are often blurred due to mixed etiology [2,4,5,6,7].

### Magnetic Stimulation and UI

The problem of UI is becoming more common due to the rising elderly population and the trend of rising prevalence of UI with aging. Deciding on a conservative or surgical treatment approach depends predominantly on the type and severity of UI and on comorbidities. Conservative treatment should be exhausted first, and, before surgery is proposed, certain factors must be taken into account: the patient’s age, general condition, and health, prior surgeries, and especially the gynecological and lower urinary tract status [4,8,9]. Therefore, new conservative treatment methods are being sought. Magnetic stimulation (MS) is a technology introduced in 1998 that has been used for stimulating the pelvic floor muscles [10]. It is based on Faraday’s law of magnetic induction, whereby a time-varying magnetic field induces electrical activity that depolarizes the nerves and causes contraction of the pelvic floor muscles. Repeated activation of the terminal motor nerve fibers and the motor end plates will tend to build muscle strength and endurance [10,11]. MS creates a rapidly pulsating magnetic field whose frequency and pulsation strength can be adjusted on the device [12]. The roots of sacral nerves S2–S4 provide the primary autonomic and somatic innervation of the urinary bladder and urethra, vaginal wall and rectum, and pelvic floor muscles. Stimulation of these roots is an efficient way to modulate the pelvic floor and subsequently control the pelvic organs [13,14]. This method is used for treating all types of urinary incontinence. MS aims to moderate the habit of frequent voiding through practicing resisting the urge to void, postponing micturition, and increasing the voiding interval, which improves the bladder capacity and decreases detrusor instability. It is painless and does not require a probe. Its advantage is that the magnetic field penetrates body tissues without significant alteration and also passes uninterrupted through clothing, and there is no need for the patient to undress [10]. The main stimulation targets in SUI are the pelvic and/or pudendal nerves, and consequently the external sphincters and/or the pelvic floor muscles. In UUI, the afferent branches of the pudendal nerve are stimulated to inhibit the detrusor muscle through central reflexes; at the same time, the efferent nerve branches are also stimulated to facilitate strengthening of the pelvic floor muscles and increase the tonus of the urethral sphincters, thereby inhibiting the detrusor muscle through the guarding reflex [15]. MS has been investigated as an alternative treatment to electrical stimulation in neurology [16,17]. It is offered as a treatment for UI, although weak evidence of the short-term and long-term effects has been found in systematic reviews. Current EUA recommendations advise against treating UI or overactive bladder (OAB) with magnetic stimulation (strength of recommendation = strong) [18].

To demonstrate the prevalence of the issues stated above, we conducted a systematic literature review. The systematic review was carried out to present recently published studies, to comprehensively evaluate the method performance, and to compare it with the results of our clinical study. Moreover, the aim of our clinical study was to determine whether the success rate of using MS to treat UI differs by UI type.

## 2. Methodology of the Systemic Review

### Search Strategy and Selection Criteria

**Figure 1:**
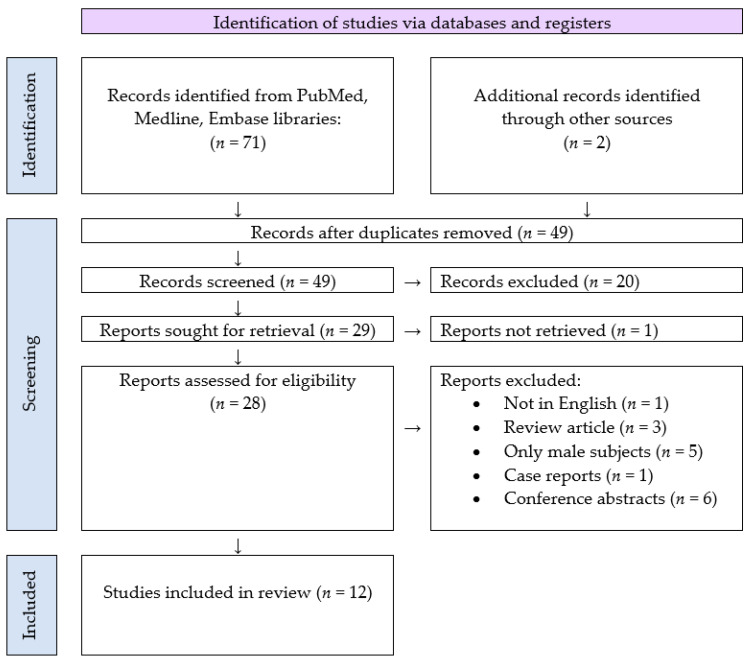
Search strategy and study selection used in this systematic review as per the PRISMA protocol.

A systematic literature search was conducted using Medline, Cochrane, and ClinicalTrials. All known synonyms were used for the following keywords: “magnetic stimulation” and “urinary incontinence”. The analysis included all clinical studies describing the evaluation of MS in the treatment of UI. All research articles in English published between 2010 and 2020 were reviewed. The article examines studies that contain the latest clinical practices for treating urinary incontinence. Potentially relevant research articles were identified by examining the abstracts or the articles as a whole. Preferred Reporting Items for Systematic Reviews and Meta-analyses (PRISMA) guidelines were used to complete the search and the article selection. Figure 1 demonstrates the PRISMA flowchart and identifies the number of search results, articles meeting criteria, and articles selected for data extraction [19]. It should also be noted that the focus of this article is only research articles. Conference presentations and reports were excluded because the goal was to focus on the most carefully evaluated material. Titles and abstracts of the identified studies were screened independently by two authors (M. Batk., T.K.). The full text of the potentially eligible studies was retrieved and independently assessed for eligibility by another author (D.L.). Any disagreement over the eligibility of particular studies was resolved through discussion with a fourth author (M.M.)

## 3. Results of the Systematic Review

Seventy-three articles were identified and screened at the title and abstract levels. Forty-five articles were excluded for any of the following reasons: they were not in English, they were review articles or meta-analyses, only males were subjects of the research, they were case reports or they were conference abstracts, or there were no possibilities for analyzing the success rate of the treatment. Thus 12 articles [20,21,22,23,24,25,26,27,28,29,30,31,32], summarized in Table 1, Table 2, Table 3, Table 4 and Table 5, represented the object of this review.

Five studies were randomized, double blinded, and sham controlled [20,21,22,23,24,32], and the rest were prospective studies without a control group [25,26,27,28,29,30,31]. Most studies used MS only for treating patients with SUI [20,21,22,23,24,25,26,31,32], whereas Samuels et al. [27], Vadala et al. [28], and Sun et al. [30] treated patients with all three types of UI: SUI, UUI, and MUI. On the other hand, Doğanay et al. [29] included patients with SUI and UUI. Our systematic review showed that the studies analyzed used different diagnostic methods to define the type and severity of UI. Initial management of patients with UI should consist of a urogynecological history with analysis of a bladder diary, urine analysis, and clinical examination. The amount and type of fluid consumed during the day should be established. The bladder diary can also be analyzed because it provides valuable information regarding the patient’s urination frequency, incontinence episodes, pad use, fluid intake, and degree of urgency and incontinence. Standardized questionnaires are sometimes used, especially to quantify symptoms; one of them is the ICIQ-UI SF. Patient history is followed by a clinical examination. Because of the high prevalence of urinary tract infections in women with lower urinary tract symptoms, urine analysis, urinary culture, and post-void residual evaluation are an indispensable part of the initial assessment of these patients. Urodynamic measurements are an important part of the diagnostic process in patients with complicated UI. The ICS specifies standard and additional urodynamic measurements. Standard measurements include uroflowmetry, post-void residual evaluation, cystometry, and pressure-flow study [1,4,5,18]. However, Table 1 shows that each study used different initial diagnostic methods and Table 3 different tools to measure outcomes. The treatment protocols were also different for each study, from six sessions to a total of 24 sessions. A long follow-up, more than 12 months, was only screened in the studies by Lim et al. [20,21] and Doğanay et al. [29].

Lim et al. [20,21] decided to use ICIQ-UI SF as the primary outcome measure based on the emerging consensus that patient-reported outcomes are the most appropriate when describing treatment success or failure. There were consistently significant improvements in the ICIQ-UI SF scores between 1 and 2 months; however, there was no further reduction of the mean ICIQ-UI SF at the 14-month follow-up in comparison to the baseline mean value of the ICIQ-UI SF. In addition to using the ICIQ-UI SF, Yamanishi et al. [22] also measured outcome with ICIQ-QOL scores and a 24-h pad test, which all decreased significantly after treatment compared to the baseline in the active treatment group. Moreover, they proved that there was no significant change from the baseline in any of the parameters in the sham treatment group. The ICIQ-UI SF was also used as the primary outcome in the study by Samuels et al. [27]. In addition, changes in the number of absorbent pads used per day were added. At the follow-up, a moderate but highly significant correlation was found between the ICIQ-UI SF score improvement and the reduction in pad usage. Vadalà et al. [28] reported that because of the small subject sample (20 patients in total), it is difficult to draw any conclusions and/or extrapolate the outcome of the study to a wider population that is experiencing UI. However, he measured the effectiveness of MS with patients’ impressions, records in urinary diaries, and scores of three life stress questionnaires (the overactive bladder symptom questionnaire [OAB-q], urinary distress inventory questionnaire-short form [UDI-6], and incontinence impact questionnaire-short form [IIQ-7]), which were performed pre- and post-treatment. Using objective methods, urodynamic tests recorded a significant increase in cystometric capacity, maximum urethral closure pressure, urethral functional length, and pressure transmission ratio values compared to the baseline values.

Weber-Rajek et al. [23], in the first study performed by her team, measured blood myostatin levels before and after MS. As outcome measurement tools, different questionnaires were added: the Revised Urinary Incontinence Scale (RUIS), the Beck Depression Inventory (BDI-II), the General Self-Efficacy Scale (GSES), and King’s Health Questionnaire (KHQ). In the following year, the same team of Weber-Rajek et al. [24] published an RCT that compared MS with pelvic floor muscle training, and the outcomes were measured with the same questionaries. In both experimental groups, a statistically significant decline in depressive symptoms (BDI-II) and an improvement in urinary incontinence severity (RUIS) and quality of life (KHQ) was seen. However, Weber-Rajek et al. [23,24] did not use the ICIQ-UI SF as a questionnaire to measure the outcome of the treatment. Özengin et al. [25] decided to compare the effectiveness of EMG-biofeedback, MS, and pelvic floor muscle training treatment. They measured the effectiveness of the treatment by evaluating pelvic floor muscles with electromyography. That study used the Incontinence Quality of Life (I-QoL) questionnaire. All three groups (the group using MS, group using EMG-biofeedback, and group performing only PFM) showed a significant improvement in EMG activity values and average QoL scores. However, the greatest improvement was observed in the EMG-biofeedback training group for QoL scores in comparison to MS and pelvic floor muscle training. In the most recent study on this topic, Silantyeva et al. [26] examined the effectiveness of MS versus pelvic floor muscle electrostimulation. In addition to the subjective evaluation with the Pelvic Floor Impact Questionnaire Short Form 7 (PFIQ-7), the researchers also used 3D ultrasound to objectively evaluate and later compare PFM anatomy and integrity. The results showed a statistically significant improvement in both subjective and objective parameters, regardless of the type of treatment; however, the results were superior in the group that underwent MS therapy. Doğanay et al. [29], who together with Lim et al. [20,21] had a longer follow-up, evaluated MS in the treatment of SUI and UUI with a 5-day voiding diary, 1-h pad test, and a validated quality of life survey (I-QOL; visual analog scale, VAS). There was statistically significant improvement in these parameters until the 1st year after the therapy, but it gradually decreased and was close to the baseline at the 3rd year after MS therapy. Bakar’s small study investigated the effectiveness of MS in the treatment of SUI before and after the therapy using pelvic floor EMG activity, a 1-h pad test, incontinence conditions utilizing VAS and quality of life using a Turkish version of the UDI-6, and the I-QoL. After MS treatment, urinary symptoms and incontinence conditions decreased, the pad test results indicated a reduction in urine loss, the EMG values also improved, and, moreover, the scores on the I-QoL, UDI-6, and VAS were lower after the treatment.

Tsai et al. [32] decided to treat refractory SUI with a magnetic coil placed directly above sacral roots S2–S4. In his sham-controlled double-blind study, the experimental group showed significant improvements in both UDI-6 and OAB-q scores after the treatment and at follow-up visits compared to the sham group. In addition, significant increases in bladder capacity, urethral functional length, and the pressure transmission ratio were also noted after the treatment. Sun et al. [30] treated patients with UI for at least 6 months following radical hysterectomy (RH) for uterine cervical cancer. There was a positive outcome after the treatment, with MS resulting in the improvement of the 1-h pad test, UDI-6, and IIQ-7, which showed statistically significant improvement. However, urodynamic parameters between pre-treatment and post-treatment after 24 sessions revealed no statistically significant changes.

A section of Table 5 points out the limitations of each study. Considering the limitations together, it can be concluded that a series of issues exists. First, further large-scale RCTs should be performed to determine consistent intervention protocols. Second, the outcome measurements to generate comparable data should be standardized. In addition, a longer follow-up period will provide more evidence to validate the effects of MS treatment. The main results from the studies analyzed confirmed that MS is effective in the treatment of UI, and similar results are also confirmed in our clinical study.

## 4. Materials and Methods of the Clinical Study

This article presents a clinical prospective non-randomized study that was carried out at the Ljubljana University Medical Center between 2016 and 2019. Patients were obtained in a urogynecology practice. It should be noted that the type of UI was previously diagnosed by a urogynecology specialist following national guidelines [4]. Before the treatment, we again evaluated the patient history, bladder diary, urine analysis, clinical examination, and the ICIQ-UI SF, and we divided them into 3 main subgroups: SUI, UUI, and MUI. The exclusion criteria included pregnancy, pacemaker patients, patients with a health condition unsuitable for performing the required measurements (hemorrhages, carcinoma, pelvic organ prolapse, inflammatory diseases, and endometriosis), and patients on antimuscarinics or beta-3 adrenergic receptor agonists.

In total, 82 consecutive patients were recruited; however, 7 patients did not provide all data. Finally, 91.4% (75) patients completed all pre- and post-treatment assessments.

The study was carried out in 3 stages. In the first stage, the patients completed a questionnaire adapted to the internationally validated ICIQ-UI SF questionnaire [33], which provides a subjective assessment of UI problems, and signed informed consent. They were informed of the possible risks. In the second stage, MS treatment was carried out, and the third stage included a checkup 3 months after the treatment was completed, during which the patients completed the same questionnaire once again (as before the treatment).

The relevant therapy program on the magnetic chair (Iskra Medical Magneto STYM^®,^ Iskra Medical d.o.o., Ljubljana, Slovenia), based on producer recommendations, was selected and is shown in Table 6. UUI patients received 20-min urgency urinary incontinence therapy, MUI patients were treated with a 20-min mixed urinary incontinence therapy, and SUI patients were treated with 20-min stress urinary incontinence therapy. The magnetic chair’s intensity can range from 0 to 100%. The electric impulse intensity was gradually increased to the patient’s tolerance level, which allowed the patient to endure a 20-min therapy session. The treatment sessions lasted 4 weeks, with 10 therapy sessions of 20-min each, and it was applied every 2 workdays.

Categorical variables were used to calculate the incidence and percentage of each factor, and all continuous variables were provided as the median and interquartile range (IQR). The normality of the data distribution was examined with the Jarque-Bera test. To understand whether pre- and post-treatment assessment of the ICIQ-UI SF scores differed based on UI type, a two-way mixed ANOVA with repeated measures was used: namely, a two-way mixed ANOVA with pre- and post-treatment as the repeated measures, and the UI type as the independent measure (pre-treatment assessment of the ICIQ-UI SF scores for MUI and UUI violated the normality assumption, and so these data were subjected to the Tukey ladder of powers transformation. Furthermore, we conducted all analyses with and without a transformation. All substantive results remained unchanged, and thus we reported the untreated solution). We tested for significant interactions: group differences in the change between pre- and post-treatment assessment of the ICIQ-UI SF scores. We performed multiple comparisons correction using Bonferroni correction. Furthermore, to assess the correlation between pre- and post-treatment assessment of theICIQ-UI SF scores, age, duration of problems, body mass index (BMI), number of births, menopause, and diabetes, Spearman’s rank correlation was used and the correlation coefficients were then interpreted following the guidelines proposed by Cohen [34], a small correlation being 0.1–0.3, medium 0.3–0.5, and large 0.5–1.0.

Before the analysis was performed, descriptive statistics were used to describe the sample. All data analyses were performed using IBM SPSS Statistics for Windows, Version 22.0, Armonk, New York, with *p* < 0.05 as statistically significant.

## 5. Results of the Clinical Study

The patients’ ages were between 42 and 92 (median 72) years. The demographics of the study sample are presented in Table 7, and descriptive statistics of the pre- and post-treatment ICIQ-UI SF scores according to UI are presented in Table 8. The study included 46.7% (35) patients with MUI, 22.6% (17) patients with SUI, and 30.7% (23) patients with UUI. Furthermore, post-treatment scores were lower than pre-treatment scores in all cases; that is, the median of pre-treatment ICIQ-UI SF scores was 16.0 for MUI (IQR: 14.0–17.0), 10.0 for SUI (IQR: 9.5–15.0), and 16.0 for UUI (IQR: 9.5–15.0), whereas the median of post-treatment ICIQ-UI SF scores was 11.0 for MUI (IQR: 9.0–16.0), 8.0 for SUI (IQR: 6.0–10.5), and 11.0 for UUI (IQR: 8.0–14.0).

To evaluate the efficacy of MS, the primary outcome of interest was considered as the change in the total score on the International Consultation on Incontinence Questionnaire (ICIQ-UI SF) score. The ICIQ-UI SF score clearly decreased following treatment of MUI, SUI, and UUI.

### 5.1. Differences between Pre- and Post-Treatment Assessment of ICIQ-UI SF Scores by UI Type

The results of the two-way mixed ANOVA showed that there was a significant main effect of UI type (F(1, 75) = 5.593, *p* = 0.005, ηp = 0.13) on pre- and post-treatment assessment of ICIQ-UI SF scores. This effect indicates that pre- and post-treatment assessment of ICIQ-UI SF scores differed by UI type.

In addition, there was a significant main effect of the pre- and post-treatment assessment of the ICIQ-UI SF scores (F(1, 75) = 102.14, *p* < 0.005 ηp = 0.577). This effect indicates that, if ignoring the patient’s UI type, the post-treatment assessment of the ICIQ-UI SF score was significantly lower (M = 10.56, SE = 0.46, 95% CI [13.18, 14.85]) compared to pre-treatment scores (M = 14.01 SE = 0.42, 95% CI [9.64, 11.48]).

**Figure 2:**
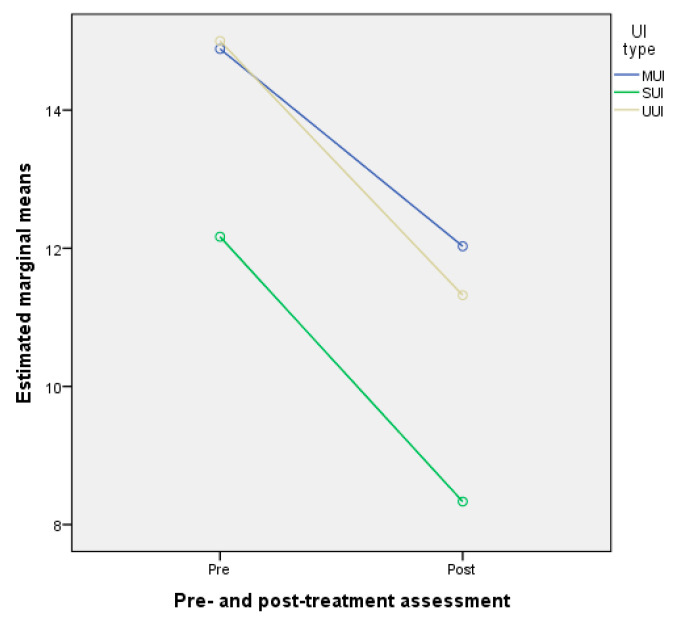
Profile plot of the two-way mixed model ANOVA marginal means of pre- and post-treatment assessment of ICIQ-UI SF scores. Abbreviations: MUI—mixed urinary incontinence, SUI—stress urinary incontinence, UUI—urgency urinary incontinence.

Moreover, Figure 2 (i.e., the profile plot of the two-way mixed model ANOVA marginal means) shows that the effect of the pre- and post-treatment assessment of the ICIQ-UI SF scores depended on the UI type. Looking at the three lines, there is a decrease in the post-assessment ICIQ-UI SF scores for all UI types. Further, looking between the lines (i.e., comparing UI types for pre- and post-treatment assessment of ICIQ-UI SF scores) shows that, among patients by UI type, compared to the pre-treatment assessment of ICIQ-UI SF scores, SUI had the greatest decrease in the post-treatment assessment of ICIQ-UI SF scores.

The two-way mixed ANOVA test was significant, and another question raised was which UI types differ from one another in the pre- and post-treatment assessment of ICIQ-UI SF scores. Answering this requires testing the differences between all pairs of UI. Therefore, we employed pairwise comparisons for the main effect of UI type corrected using Bonferroni adjustments. The results showed a significant difference (*p* < 0.01) between MUI and SUI and between SUI and UUI, but not between MUI and UUI (*p* > 0.05).

As predicted, patients with the MUI type had lower improvement in their post-test ICIQ-UI SF score compared to SUI (*p* = 0.006), and those with UUI had lower improvement in their post-test ICIQ-UI SF score compared to SUI (*p* = 0.024).

### 5.2. Correlation between Pre- and Post-Treatment Assessment ICIQ-UI SF Scores by UI Type

The Spearman rank correlation was also calculated between participants’ demographics and pre- and post-treatment assessment of ICIQ-UI SF scores by UI type. There was only a moderate statistically significant correlation between BMI and the post-treatment assessment of ICIQ-UI SF scores for the MUI type (rs = 0.416, *p* = 0.01) and a moderate statistically significant correlation between BMI and the post-treatment assessment of ICIQ-UI SF scores for the UUI type (rs = 0.415, *p* = 0.04).

The correlation was also assessed between intensity, improvement (difference between pre- and post-treatment assessment of ICIQ-UI SF scores), and BMI. Only a medium positive correlation was found between intensity and BMI, which was statistically significant, with rs = 0.277, *p* = 0.014, which means that a higher intensity is associated with a higher BMI.

## 6. Discussion

The aim of this study was to assess and analyze the effectiveness of MS in the treatment of female urinary incontinence. On the basis of the results, it can be observed that UI improved after treatment with MS. The ICIQ-UI SF score improved in patients regardless of the type of UI. However, the greatest decrease in the post-treatment assessment of the ICIQ-UI SF score was among patients with SUI. Moreover, the results also showed that UI type had a statistically significant effect on the post-treatment score of both MUI and SUI but not on UUI.

We treated patients three times a week for 4 weeks, with 10 sessions altogether. Galloway et al. [12] reported that patients were treated twice a week for 6 weeks and MS significantly improved SUI. Yamanishi et al. [35] reported that MS of the pelvic floor twice weekly for 5 weeks significantly improved SUI as well as UUI. In another study, Yokoyama et al. [36] treated female patients twice a week for 8 weeks with the same results. The same ameliorating results were found by Özengin et al. [25], who compared three different treatment methods for SUI: MS, EMG biofeedback, and PFMT. In that study, a statistically significant improvement in PFMT activity was noted in all three treatment groups, with no statistical differences between groups. They concluded that MS is a highly user-friendly modality for the conservative treatment of female UI. In the most recent study on this topic, Silantyeva et al. [26] examined the effectiveness of MS versus electrostimulation after short-term therapy (10 sessions) in postpartum women of childbearing age who had had a vaginal delivery in the previous 6 months. In addition to the subjective evaluation, a 3D ultrasound was used to objectively evaluate and later compare the PFM anatomy and integrity. The results showed a statistically significant improvement in both subjective and objective parameters, regardless of the type of treatment; however, the results were superior in the group that underwent MS therapy. The authors attribute this to the ability of the magnetic field to penetrate deep into pelvic tissues and therefore uniformly activate PFM, whereas electrical stimulators lose a major portion of the energy released on the tissue surface and the PMF are activated with a smaller intensity.

Essentially, each study in the review and the conclusion of the prospective study had a limitation of sorts. For clarifying the impact on the extent of amelioration after therapy with MS, the parameters of stimulation should be unified with regard to timeframe, impulse intensity, and follow-up tracking. Among the limitations, the most common were a relatively small study sample, which significantly decreases the statistical relevance of the study, and the lack of long-term evaluation of the patients. Only five studies [20,21,22,23,24,32] included a control group, and six of them [20,21,23,25,26,30,32] did not have clear treatment protocols, refined study parameters, and/or objective instruments or measurements to evaluate the results. Furthermore, the potential placebo effect of the sham stimulators was not analyzed in any study.

There are also some limitations to the analysis that should be considered when interpreting these results. First, and perhaps most important, our sample was non-randomized. Although this non-probability sampling method is the most applicable and widely- used method in clinical research [37], the sampling method does not guarantee equal chances for each subject in the target, it is less representative of the target population, and it decreases the ability to draw completely impartial conclusions about MS effectiveness. Second, the power of our study was low, as well as the power of most studies in our systematic review (Table 2). An ideal study is one that has high power. This means that the study has a high chance of detecting a difference between groups if it exists, and consequently, if the study demonstrates no difference between groups, the researcher can be reasonably confident in concluding that none exist. According to the literature review, the ideal power for any study is considered to be 80% [38]. For our study to achieve a significance level of 95% and a power of 80%, the sample size should equal 189; in our study, the sample size of 76 accounted for a power of 57% [39]. This means that our study had low power, and studies with lower power increase the likelihood that a statistically significant finding represents a false positive result. Future studies may address all of the above limitations and test the robustness of these results on an extended environment. One limitation could also be that our study included only the ICIQ-UI SF as the tool for measuring the effectiveness of MS in the treatment of UI. However, this questionnaire is the only available validated questionnaire in Slovenian [33]. We are convinced that patient-reported outcomes are the most appropriate when describing treatment success or failure. As we also concluded in the systematic review, we are aware that outcome measurements to generate comparable data should be standardized.

Nevertheless, we assume that this method of treatment has future potential. Last but not least, the population is aging and increasingly more patients are seeking more user-friendly treatment modalities and avoiding surgeries. Behavioral therapy and all the efforts to educate patients and encourage successful management strategies and guarding techniques will serve to promote optimal outcomes and achieve durable benefits [9,12,40,41,42]. We must be aware that, beyond technical parameters, the improvement of QoL after MS treatment is undoubtedly associated with social predictors (e.g., age, sex, rural living, number of household members, and financial problems) and not only clinical predictors (e.g., disease severity, disability, disease duration, motor impairment, depressive symptoms, complications of therapy, and gait impairment). The success of treatment varies according to the severity of the muscle weakness before treatment. The same statement and conclusions were drawn by Lim et al. [20], who noticed the lack of randomized, sham-controlled trials and the lack of recommendations on the use of MS for the conservative treatment of female UI.

## 7. Conclusions

Regardless of the limitations of and variations between the studies examined here, some universal conclusions can be drawn. Namely, MS is a non-invasive treatment method that effectively and safely improves quality of life by promoting urinary continence in women that experience refractory UI. These patients, who are perhaps not motivated to perform regular PFM strengthening exercises, can be conservatively treated. The results after MS treatment show a reduction in the number of daily leakages and pad usage, and therefore a reduced number of incontinence episodes. This is a painless and comfortable method, with good compliance by patients. Additional advantages include no side effects, no need to undress, and automatic contractions.

We conclude that future studies are necessary, all of which should include a large sample size, a control group, an optimal research protocol, pre-treatment analyses, standardization, and longer follow-ups. Relevant conclusions, which could be drawn only from a well-performed study with longer observation periods and cost-benefit analysis, would have a major impact on defining the applicability of MS and standardizing its use in clinical practice as a widespread, non-invasive treatment method for patients with mild to moderate SUI and eventually for other types of UI.

## Figures and Tables

**Table 1:** Clinical overview of 12 articles: study type and diagnostic method(s).

Study	Study Type	Diagnostic Method(s)
Lim et al., 2015, 2017 [20,21]	Multicenter, randomized, double-blind, sham-controlled (1:1 ratio)	Urinary diary ICIQ-UI SF Perineometer PGI-I ICIQ-LUTSqol 1-h pad test
Yamanishi et al., 2017 [22]	Pilot, randomized, sham-controlled (active: sham = 2:1)	7-day urinary diary 24-h pad test ICIQ-UI SF ICIQ-LUTSqol Urodynamic test
Weber-Rajek et al., 2019 [23]	Randomized, double-blind, controlled pilot study	Voiding diary QUID Patient history
Weber-Rajek et al., 2020 [24]	Randomized, double-blind, controlled trial	SUI previously diagnosed by urologist Voiding diary Patient history
Özengin et al., 2016 [25]	Open-label, not controlled, data on randomization N/A	SUI previously diagnosed by gynecologist EMG-BF measurement of PFM activity at rest and during three maximum voluntary contractions
Sylantieva et al., 2020 [26]		3D transperineal ultrasound Patient history
Samuels et al., 2019 [27]	Prospective, multi-center, open-label, single-arm study; data on randomization N/A	Reporting UI symptoms ICIQ-UI SF
Vadalà et al., 2017 [28]	Retrospective observational study	Previously diagnosed UI Voiding diary Urodynamic examination Urinalysis
Doğanay 2010 [29]	Prospective, open-label, single-center, not controlled	5-day voiding diary Urodynamic studies I-QOL VAS
Sun et al., 2014 [30]	Open-label, single-center, not controlled	Urodynamic studies after RH UDI-6 IIQ-7
Bakar et al., 2010 [31]	Open-label, single-center	Previously diagnosed SUI 1-h pad test
Tsai et al., 2014 [32]	Sham-controlled, double-blind, parallel study	Urodynamic studies U-UDI OAB-q

**Table 2:** Clinical overview of 12 articles:UI type, sample size, control, length of intervetion period and frequency.

Study	UI Type	Sample Size	Control Group	Length of Intervention Period and Frequency
Lim et al., 2015, 2017 [20,21]	SUI	120	Yes, 1:1 ratio	20 min stimulation twice a week for 8 weeks (16 sessions total)
Yamanishi et al., 2017 [22]	SUI	39	Yes, active stimulation and sham stimulation group, 2:1 ratio	20 min stimulation once a week for 10 weeks (10 sessions total)
Weber-Rajek et al., 2019 [23]	SUI	52	Yes, active: control = 28:24	15 min stimulation three times a week for 4 weeks (12 sessions total)
Weber-Rajek et al., 2020 [24]	SUI	111	Yes, PFMT (40), MS (37), control (34)	PFMT: 45 min sessions three times a week for 4 weeks (12 sessions total)
Özengin et al., 2016 [25]	SUI	67	No	MS: 20 min sessions three times a week for 8 weeks (24 sessions), EMG-BF: three sessions of 20 min in 8 weeks, PFMT: N/A
Sylantieva et al., 2020 [26]	SUI?	95	Yes	MS: 28 min sessions two to three times a week (10 sessions total)
Samuels et al., 2019 [27]	SUI (49%), UUI (11%), MUI (40%)	75	No	28 min twice a week, for 3 weeks (six sessions total)
Vadalà et al., 2017 [28]	SUI (50%), UUI (20%), MUI (30%)	20	No	20 min stimulation twice a week for 3 weeks (six sessions total)
Doğanay 2010 [29]	SUI, UUI	137; SUI: 68, UUI: 69	No	20 min sessions twice a week for 8 weeks (16 sessions total)
Sun et al., 2014 [30]	SUI, UUI, MUI	32	No	20 min sessions twice a week for 12 weeks (24 sessions total)
Bakar et al., 2010 [31]	SUI	13	No	20 min sessions twice a week for 6 weeks (12 sessions total)
Tsai et al., 2014 [32]	SUI	30	Yes, active group: 18, sham group: 12	20 min every weekday for 12 days (12 sessions total)

**Table 3:** Clinical overview of 12 articles: device and outcomes (changes).

Study	Device	Outcomes (Changes)
Lim et al., 2015, 2017 [20,21]	QRS-1010 PelviCenter (QRS International, Ruggell, Liechtenstein)	ICIQ-UI SF reduction (total and QoL) Cure (objective and subjective) SUI symptoms (IEF, 1-h pad test, PFM examination with perineometer, incontinence severity)
Yamanishi et al., 2017 [22]	Armchair-type magnetic stimulator (Nihon Kohden, Tokyo, Japan)	Number of incontinence episodes per week (frequency) Degree of UI (using 24-h pad test) ICIQ-UI SF reduction (total and QoL) ALPP in urodynamic studies
Weber-Rajek et al., 2019 [23]	NeoControl chair (Neotonus Inc., Marietta, GA, USA)	Myostatin concentration (before and after treatment) RUIS BDI
Weber-Rajek et al., 2020 [24]	MS: NeoControl chair (Neotonus Inc., Marietta, GA, USA)	Better RUIS scores 2. KHQ 3 GSAS (in ExMI group)
Özengin et al., 2016 [25]	N/A	QoL (in all groups) EMG activity (in all groups)
Sylantieva et al., 2020 [26]	N/A	PFM thickness PFDI-20
Samuels et al., 2019 [27]	BTL EMSELLA (BTL Industries Inc., Boston, MA, USA)	ICIQ-UI SF Pad usage (urine leakage)
Vadalà et al., 2017 [28]	Magneto STYM (Iskra Medical, Ljubljana, Slovenia)	Number of incontinence episodes and nocturia Urodynamic testing results Life stress scores
Doğanay 2010 [29]	Neotonus Inc., (Marietta, GA, USA)	5-day voiding diary 1-h pad test I-QoL VAS (no statistically significant changes in urodynamic parameters)
Sun et al., 2014 [30]	BioCon-2000WTM, Mcube Technology Co. (Korea)	UDI-6 IIQ-7 1-h pad test (no statistically significant changes in urodynamic parameters)
Bakar et al., 2010 [31]	EMD, E-6000 MAGTHER, TR	1-h pad test, pelvic floor EMG, VAS, UDI-6, I-QoL
Tsai et al., 2014 [32]	Magstim Rapid2 and a 70 mm figure-8 coil/sham coil	U-UDI Urodynamic values

**Table 4:** Clinical overview of 12 articles: follow-up period and benefits.

Study	Follow-Up Period	Benefits (Statistically Significant)
Lim et al., 2015, 2017 [20,21]	1, 2, 5, 8, 14 months	Treatment response (regardless of number of MS sessions) and positive outcomes after MS therapy Reduction in ICIQ-UI SF post-treatment score at all follow-ups
Yamanishi et al., 2017 [22]	10 weeks	Amelioration of signs and symptoms (subjective and objective evaluation) No device or study-related side effects
Weber-Rajek et al., 2019 [23]	4 weeks	Reduction of: UI severity, Myostatin level, Subjective depression level
Weber-Rajek et al., 2020 [24]	4 weeks	Improved general quality of life after both MS and PFMT Reduction of SUI symptoms after ExMI and PFMI Subjective depression level
Özengin et al., 2016 [25]	8 weeks	Improved quality of life in all groups, with superior improvement in EMG-BF group Improved PFM EMG activity after three types of treatment (no statistical differences between groups)
Sylantieva et al., 2020 [26]	4 weeks	Improvement of biometric indices of pelvic floor integrity Profound change in PFDI-20, especially in MS group Substantially greater improvement of subjective intimate health (self-evaluation bimodal questionnaire) in MS compared to ES group
Samuels et al., 2019 [27]	3 weeks, 3 months	Improved UI symptoms Improved quality of life Less leakage Use of pads halved
Vadalà et al., 2017 [28]	3 weeks	Reduction of micturition frequency and nocturia Improved urodynamic testing results (increased cystometry capacity, MUCP, urethral functional length, PTR) No side effects reported General satisfaction
Doğanay 2010 [29]	2, 4, 6, 8 weeks, 6 months, 1, 2, 3 years	Subjective and objective improvement of symptoms in women with SUI and UUI ExMI effect lasted about 1 year, then gradually decreased
Sun et al., 2014 [30]	4 weeks, 12 weeks	Objective and subjective symptom amelioration
Bakar et al., 2010 [31]	6 weeks	Improvement of subjective symptoms Better EMG results
Tsai et al., 2014 [32]	18 weeks (4.5 months)	Significant increase in bladder capacity, urethral functional length, and pressure transmission ratio U-UDI Higher therapeutic frequency within shorter time period can produce greater cumulative effect that most benefits patients with SUI

**Table 5:** Clinical overview of 12 articles: limitations.

Study	Limitations
Lim et al., 2015, 2017 [20,21]	Possible placebo effect in sham group Long follow-up period possible No urodynamic testing performed (reason for SUI: hypermobility and/or intrinsic sphincter deficiency is unknown)
Yamanishi et al., 2017 [22]	Small sample size (insufficient statistical power) Low frequency of treatments per week (once a week) Only short-term effects evaluated Possible biased results because of PFMT before MS, although patients were refractory to PFMT
Weber-Rajek et al., 2019 [23]	Relatively small study sample No long-term effect evaluation
Weber-Rajek et al., 2020 [24]	No objective instruments/measurements included No long-term evaluation
Özengin et al., 2016 [25]	No long-term follow-up Relatively small sample size Only one objective instrument used (EMG)
Sylantieva et al., 2020 [26]	Only young subjects (of reproductive age) included No long-term follow-up Exact treatment protocols not described Detailed diagnostic criteria not enlisted
Samuels et al., 2019 [27]	No control group Relatively short follow-up period
Vadalà et al., 2017 [28]	Very small sample size (insufficient statistical power) of different types of UI, 2. No follow-up period
Doğanay 2010 [29]	Lack of placebo/sham group
Sun et al., 2014 [30]	Lack of placebo/sham group Small sample size No pre-surgery evaluation of incontinence No long-term follow-up
Bakar et al., 2010 [31]	Very small sample size, No long-term follow-up, Lack of control group, Only older demographic included
Tsai et al., 2014 [32]	Small sample size Non-refined study parameters No long-term follow-up

**Table 6:** Urinary incontinence treatment programs.

Programs	Step	Frequency of Magnetic Stimulation	Time	Active Time	Pause Time	Therapy Time
UUI	1/1	10 Hz	12 s	6 s	6 s	20 min
SUI	1/1	35 Hz	12 s	6 s	6 s	20 min
MUI	1/2	10 Hz	12 s	6 s	6 s	10 min
	2/2	35 Hz	12 s	6 s	6 s	10 min

**Table 7:** Participants’ demographics.

Variable	UI Type		
	MUI (*n* = 35)	SUI (*n* = 17)	UUI (*n* = 23)
Age (years)			
Median	73.0	63.0	73.0
IQR (Q1–Q3)	15.0 (14.0–17.0)	21.0 (14.0–17.0)	12.0 (14.0–17.0)
Duration of problems (years)			
Median	6.0	6.0	5.0
IQR (Q1–Q3)	7.0 (3.0–10.0)	19.5 (3.0–22.5)	8.0 (2.0–10.0)
BMI (kg/m^2^)			
Median	25.7	23.6	26.0
IQR (Q1–Q3)	10.3 (21.6–31.9)	4.0 (22.3–26.3)	7.4 (24.2–31.6)
Menopause (% yes)	30 (85.7)	13 (76.5)	21 (92.9)
Diabetes (% yes)	1 (2.9)	0 (0.0)	5 (20.0)
Previous gynecological surgeries (% yes)	16 (45.7)	5 (27.8)	11 (48.0)

**Table 8:** Descriptive statistics for pre- and post-treatment of ICIQ-UI SF scores by UI type.

Variable	UI Type		
	MUI (*n* = 35)	SUI (*n* = 17)	UUI (*n* = 23)
Pre-treatment (ICIQ-UI SF score)			
Median	16.0	10.0	16.0
IQR (Q1–Q3)	3.0 (14.0–17.0)	5.5 (9.5–15.0)	3.0 (13.0–16.0)
Post-treatment (ICIQ-UI SF score)			
Median	11.0	8.0	11.0
IQR (Q1–Q3)	7.0 (9.0–16.0)	4.5 (6.0–10.5)	6.0 (8.0–14.0)

## Data Availability

The data presented in this study are openly available with the author.
